# Fibrinogen Chains Intrinsic to the Brain

**DOI:** 10.3389/fnins.2019.00541

**Published:** 2019-05-29

**Authors:** Eugene V. Golanov, Martyn A. Sharpe, Angelique S. Regnier-Golanov, Gregory J. Del Zoppo, David S. Baskin, Gavin W. Britz

**Affiliations:** ^1^Department of Neurosurgery, Houston Methodist Hospital, Houston, TX, United States; ^2^Division of Hematology, University of Washington School of Medicine, Seattle, WA, United States

**Keywords:** fibrinogen, brain, astrocytes, neurons, subarachnoid hemorrhage, intrinsic, fibrinogen chain, fibrin

## Abstract

We observed fine fibrin deposition along the paravascular spaces in naive animals, which increased dramatically following subarachnoid hemorrhage (SAH). Following SAH, fibrin deposits in the areas remote from the hemorrhage. Traditionally it is thought that fibrinogen enters subarachnoid space through damaged blood brain barrier. However, deposition of fibrin remotely from hemorrhage suggests that fibrinogen chains Aα, Bβ, and γ can originate in the brain. Here we demonstrate *in vivo* and *in vitro* that astroglia and neurons are capable of expression of fibrinogen chains. SAH in mice was induced by the filament perforation of the circle of Willis. Four days after SAH animals were anesthetized, transcardially perfused and fixed. Whole brain was processed for immunofluorescent (IF) analysis of fibrin deposition on the brain surface or in brains slices processed for fibrinogen chains Aα, Bβ, γ immunohistochemical detection. Normal human astrocytes were grown media to confluency and stimulated with NOC-18 (100 μM), TNF-α (100 nM), ATP-γ-S (100 μM) for 24 h. Culture was fixed and washed/permeabilized with 0.1% Triton and processed for IF. Four days following SAH fibrinogen chains Aα IF associated with glia limitans and superficial brain layers increased 3.2 and 2.5 times (*p* < 0.05 and *p* < 0.01) on the ventral and dorsal brain surfaces respectively; fibrinogen chains Bβ increased by 3 times (*p* < 0.01) on the dorsal surface and fibrinogen chain γ increased by 3 times (*p* < 0.01) on the ventral surface compared to sham animals. Human cultured astrocytes and neurons constitutively expressed all three fibrinogen chains. Their expression changed differentially when exposed for 24 h to biologically significant stimuli: TNFα, NO or ATP. Western blot and RT-qPCR confirmed presence of the products of the appropriate molecular weight and respective mRNA. We demonstrate for the first time that mouse and human astrocytes and neurons express fibrinogen chains suggesting potential presence of endogenous to the brain fibrinogen chains differentially changing to biologically significant stimuli. SAH is followed by increased expression of fibrinogen chains associated with glia limitans remote from the hemorrhage. We conclude that brain astrocytes and neurons are capable of production of fibrinogen chains, which may be involved in various normal and pathological processes.

## Introduction

Arrest of cerebrospinal fluid (CSF) flow can be triggered by subarachnoid hemorrhage (SAH). Following SAH, fibrin is deposited in the paravascular space hindering CSF flow (Gaberel et al., 2014; Siler et al., 2014; Golanov et al., 2017). The blockage of CSF flow can be partially reversed by rt-PA or anti-tissue factor (TF, FIII) antibodies, suggesting that fibrin deposition in subarachnoid space (SS) may play an important role in CSF arrest (Gaberel et al., 2014; Siler et al., 2014; Golanov et al., 2017).

Astrocyte endfeet form the glia limitans on the brain surface placing astrocytes, a primary source of TF in the brain (Eddleston et al., 1993), in direct contact with blood following SAH. Activation of factor VII to factor VIIa in the blood and its interaction with TF (to form the TF: VIIa complex), initiate the extrinsic pathway of coagulation that ultimately convert prothrormbin to thrombin and hence promote fibrin formation.

Fibrinogen is not considered to be present in the brain, unless there is breach of the cerebral vasculature blood-brain or permeability barrier (BBB) and leakage of plasma/blood into the neuropil or fluid spaces (Akassoglou and Strickland, 2002; Adams et al., 2004; Petersen et al., 2018). Levels of fibrinogen in the CSF are very low, ∼0.65 μg/ml (Thompson, 2005), especially compared to the blood levels of fibrinogen (2–3 mg/ml) (Redman and Xia, 2001; Colman and Budzynski, 2011).

Blood coagulation is considered to participate in certain intravascular immunity responses targeted toward immobilization of the foreign agent (Doolittle, 2011, 2016; Gaertner and Massberg, 2016), and thus is a part of acute phase response (Gruys et al., 2005; Cray et al., 2009). Fibrinogen and prothrombin are necessary components of both intrinsic and extrinsic coagulation pathways. Fibrinogen is a 340 kDa glycoprotein consisting of three dimers. Each dimer is formed by identical polypeptides formed by fibrinogen chains Aα, Bβ, and γ, respectively. Fibrinogen is assembled in the endoplasmic reticulum of hepatocytes with the help of chaperon proteins, contains 29 disulfide bonds, and after glycosylation is released into the circulation (Redman and Xia, 2001; Mosesson, 2005; Weisel, 2005). Each of the three polypeptide chains forming fibrinogen is encoded by the respective genes, FGA, FGB, and FGG. It is thought that these genes are paralogs and are common for extant vertebrates (Doolittle et al., 1997). Thrombin, serine protease, which is present in brain (Deschepper et al., 1991; Dihanich et al., 1991) cleaves fibrinogen releasing fibrinopeptide A and B and initiating of fibrin polymerization and fibrin gel formation (Weisel, 2005).

Earlier observations and our recent experiments (Golanov et al., 2017) suggest that fibrin in the subarachnoid/paravascular space present in naïve animals and is being deposited in the areas remote from the hemorrhage following the SAH (Suzuki et al., 1977). Fibrin(ogen) was observed intracellularly (Marik et al., 2007; Yates et al., 2017) under pathological conditions such as multiple sclerosis. It is generally accepted that BBB insufficiency leads to blood-born fibrinogen penetration into the brain and consequent formation of fibrin depositions accompanying neurological disorders (Petersen et al., 2018).

The majority of circulating fibrinogen is being synthesis in liver (Tennent et al., 2007). However, fibrinogen can also be synthesized in extrahepatic sites such as bone marrow, lungs, intestine epithelial cells (see Weisel, 2005). We hypothesize that non-vascular brain cells, including astroglia and, probably, neurons, are capable of expressing fibrinogen chains Aα, Bβ, and γ, which, in turn, may exert multiple effects in the brain in normal and pathological conditions. Here we provide *in vivo* and *in vitro* data that support this hypothesis.

## Materials and Methods

All animal procedures were conducted in accord with the U.S. National Institutes of Health “Guide for the care and use of laboratory animals” and approved by the Institutional Animal Care and Use Committee of Houston Methodist Research Institute, Houston, TX, United States. Animals were housed in the institutional animal facilities on 12 h day/night cycle with *ad libitum* access to food and water. Studies were performed in sixteen 10–14 weeks old male C56BL/6J mice (Jackson Labs).

### General Procedures

Anesthesia was induced by placing animals in a closed chamber flushed with 3% isoflurane in air; after reaching sufficient depth of anesthesia the animals were maintained at depth by 1.5–1.75% isoflurane in 80%/20% N2/O2 mixture delivered by facemask. Core body temperature was maintained at 37°C using a thermoblanket with the feedback from the rectal probe.

### SAH in Mice

Subarachnoid hemorrhage was triggered by monofilament perforation of the Circle of Willis as previously described (Bederson et al., 1995; Parra et al., 2002; Feiler et al., 2010). Briefly, animals were anesthetized and placed in the supine position. The left common, external and internal carotid arteries were exposed through the midline incision. All three arteries were mobilized using silk sutures (7-0). The stump was formed from the external carotid artery by cutting it between two ligatures. A proline monofilament (6-0) was advanced into the internal carotid artery the stump until slight resistance was felt. The filament was advanced further forward for ∼1 mm and withdrawn. The stump was ligated, the wound was closed, and the animal was allowed to recover. In the sham group, the filament was advanced to the resistance point and withdrawn without perforation. After surgery, the animals were allowed to recovery and then returned to the home cage.

### Immunohistochemistry

Four days after SAH (or sham operation) the mice were deeply anesthetized and sacrificed by transcardiac perfusion with saline followed by 4% paraformaldehyde in PBS. Brains were removed, post-fixed for 24 h, and cryoprotected in 30% sucrose in PBS until complete submersion. The brains were quick frozen on dry ice, embedded in mounting media (NEG50, Richard-Allan Scientific, Thermo Scientific), and stored at −80°C until further processed. Ten micrometer thick coronal sections were prepared using a cryotome (Microm HM550, Thermo Scientific) and collected on glass slides. The glass slides with samples were washed in PBS-0.3% Triton X-100 for 30 min at room temperature, rinsed in distilled water, and air dried. A paraffin pen was used to contour the brain slices on the glass slides (ImmEdge Pen, Vector Laboratories). After the slides were rinsed in PBS to re-wet tissue, the sections were blocked with 2% normal donkey serum (Jackson ImmunoResearch) in PBS-0.3% Triton X-100 for 2 h. After blocking, the slides were incubated overnight (12–14 h) with the primary specific antibodies against fibrinogen-α, −β and −γ chains (1:200; rabbit polyclonal; Proteintech). The slides were washed three times for 15 min in PBS-0.3% Triton X-100, and then incubated with secondary antibodies for 3 h (donkey anti-rabbit-Alexa Fluor^®^ 488 and Alexa Fluor^®^ 594; 1:1000; Life Technologies, Thermo Fisher Scientific). The slides were rinsed in PBS and distilled water, covered with protective Fluorescent Mounting Medium with DAPI (GBI Labs), glass covered, and sealed with nail polish. In control slides, primary antibodies were omitted.

### Cell Cultures

Normal human astrocytes (NHA) were obtained from Lonza (Walkersville, MD, United States) and human cortical neurons (HCN) from the American Type Culture Collection (ATCC, Manassas, VA, United States) and grown subject to vendor recommendations. NHA were grown in Astrocyte Basal Medium supplemented with 3% FBS, glutamine, insulin, fhEGF, GA-1000, and ascorbic acid. HCN were prepared using ATCC-formulated Dulbecco’s modified Eagle’s medium (cat# 30-2002) and supplemented with 10% FBS. HCN and NHA were grown to confluence in the appropriate media on Costar 96-well growth plates (Corning, NYC, NY, United States), and were grown for 24 h in the presence/absence of all effectors, in a total volume of 250 μL. The cells were fixed in ice-cold 4% PFA, were washed/permeabilized with 0.1% Triton X-100 in PBS, and then treated with Dako protein blocking solution. The cells were then incubated in 0.1% avidin (Sigma) for 30 min, washed and then incubated 10 μM biotin for 15 min, and washed in PBS.

The following primary antibodies were used: rabbit polyclonal fibrinogen chain Aα antibody ([H-300] (Santa Cruz Biotechnology, Santa Cruz, CA, United States), fibrinogen chain Bβ antibody ([H-270] Santa Cruz), fibrinogen chain γ antibody ([H-194] Santa Cruz) and mouse monoclonal antibodies to GFAP ([2A5] Santa Cruz). The following secondary antibodies were used: Donkey Anti-Mouse IgG H&L (Alexa Fluor^®^ 488) (ab150105) and (Alexa Fluor^®^ 594) (ab150108), Donkey Anti-Rabbit IgG H&L (Alexa Fluor^®^ 488) (ab150073) and (Alexa Fluor^®^ 594) (ab150076), all from Abcam (Abcam, Cambridge, MA, United States). All antibodies were diluted using Dako antibody diluent (S3022) at 1:100 for the primary antibodies and 1:250 for the secondary antibodies. Cell nuclei were labeled using DAPI.

### Culture Stimulation

Normal human astrocytes and neurons grown to confluence were stimulated with the nitric oxide donor, NOC-18 (100 μM; Santa Cruz, CA, United States), tumor necrosis factor alpha, TNF-α (100 nM; Life Technologies, Carlsbad, CA, United States), or the stable ATP analog, ATP-γ-S (100 μM, Sigma, MS) for 24 h. The cultures were fixed and washed/permeabilized with 0.1% Triton and processed for quantitative immunofluorescence.

### Fluorescent Imaging

Fluorescent images were captured using a Nikon Eclipse TE2000-E at 4×, 20×, or 30× magnification using a CoolSnap ES digital camera system (Roper Scientific) containing a CCD-1300-Y/HS 1392-1040 imaging array that is cooled by Peltier. Images were recorded and analyzed using Nikon NIS-Elements software (Elements 3.22.11). All images were saved as JPEG2000 files using Nikon NIS-Elements. In culture, cell numbers in the field of view in one well were counted manually, using DAPI nuclei fluorescence, and after background removal, fluorophore levels were calculated as signal/cell. Statistical analysis consisted of ANOVA followed by t-test using Bonferroni multiple-comparison correction.

To evaluate the intensity of fluorescence in the brain tissue, the fluorescent images of the ventral and dorsal regions of interest (ROI) were taken at 20x using standard exposure time by blinded to the experiment observer and processed simultaneously. Using ImageJ software color channels were split and equally thresholded after subtraction of the background and converted into binary images (Iliff et al., 2012). Five ROI were measured in each animal and fluorophore levels were calculated followed by statistical analysis using ANOVA followed by Bonferroni multiple comparison. All data expressed as mean ± SEM.

### Human Brain Tissue Sections

Human brain tissue samples were procured, after obtaining signed informed consent and institutional review, from patients undergoing surgically indicated resection of malignant gliomas at the Houston Methodist Hospital. Tissue was collected directly from the operating room, given an identifying code to ensure patient confidentiality, and fixed in ice-cold 4% paraformaldehyde (PFA) within 15 min of removal. For details see Sharpe and Baskin (2016).

The tissue microarray block was sliced into 5-μm sections that were affixed to slides and dried. Slides were dewaxed four times in xylene, twice in isopropanol, and rehydrated using graded ethanol. The slides were washed and permeabilized using PBS (Fisher Scientific, Waltham, MA, United States) containing 0.1% Triton X-100. Epitope retrieval was done by heating the slides in Na-citrate buffer (100 mM, pH 6.0) at < 100°C for 30 min, followed by cooling slowly to bench temperature. After washing in PBS, endogenous peroxidase activity was eliminated using mild conditions: 1.8% H2O2 for 5 min, 1% Periodate for 5 min, 0.02% NaBH4 for 2 min. The slides were blocked using Serum-Free Protein Block (Dako North America, Inc., Carpinteria, CA, United States) and incubated with primary antibodies (same that were used for mouse tissue processing) overnight at room temperature, at 1:100 dilution. After washing in PBS, the HiDefTM HRP-polymer system (Cell Marque, Rocklin, CA, United States) was used to functionalize with peroxidase, and visualization was performed using the Dako DAB chromogen kit according to manufacturer’s guidelines. Additional slides were also incubated with either hematoxylin/100 mM LiOH to render the nuclei blue for visualization of cells using transmission microscopy or 0.1% cresyl violet solution for 3 min, followed by washing (Nissl Staining).

### Western Blot

Normal human astrocytes cell cultures were collected at confluence and processed for protein extraction. Cell pellets were lysed with RIPA buffer (Thermo Fisher Scientific) by pipetting them up and down and then placed for 30 min on an orbital shaker at 4°C. Proteins were quantified by Pierce BCA Protein Assay Kit (Thermo Fisher Scientific) and resolved by 4–15% SDS-PAGE Gel (Bio-Rad) according to the manufacturer’s instruction.

Antibodies used were: rabbit anti-fibrinogen-Aα, anti-fibrinogen-Bβ and γ (1:1000, respectively H-300, H-270 and C-20; Santa Cruz Biotechnologies, Santa Cruz, CA, United States); anti-goat-HRP (1:2000; 81-1620; Thermo Fisher Scientific) and anti-rabbit-HRP (1:2000, 1706515; Bio-Rad).

### RNA Extraction and RT-qPCR

Total RNA was extracted from NHA cell cultures (*n* = 3) using Aurum Total RNA mini kit (Bio-Rad). RNA concentration was determined using a NanoDrop ND-1000 spectrophotometer (NanoDrop, Rockland, DE, United States). Quantitative RT–PCR was run with 20ng of RNA using the 1-step Rt-qPCR mix from Bio-Rad according to the supplier’s protocol. Amplification was done using SYBR green chemistry on CFX96 Real-Time PCR System (Bio-Rad). Each sample was run in duplicate. PCR primers were designed with primer designing tool from NCBI and manufactured by Sigma. Hprt was used as the reference gene. The following primers’ sequences were used: FibA-α, 5′-AGA CAT CAA TCT GCC TGC AAA-3′ and 5′-AGT GGT CAA CGA ATG AGA ATC C-3′; FibB-β, 5′-GTC CCA AGG TGT CAA CGA CAA-3′ and 5′-CTC TCT TCT TGT CAA GGG GTC G-3′; Fib-γ 5′-TTA TTG TCC AAC TAC CTG TGG C-3′ and 5′-GAC TTC AAA GTA GCA GCG TCT AT-3′; HPRT, 5′-CCT GGC GTC GTG ATT AGT GAT-3′ and 5′-GGG CTA CAA TGT GAT GGC CT-3′.

### Down-Regulation of the FibA-α, FibB-β, and Fib-γ in NHA Cells by siRNA

FGA siRNA (assay# s5115), FGB siRNA (s5119) FGG siRNAs (s5179) and negative control #1 (#4390843) were obtained from Thermo Fisher Scientific. Each siRNA concentration was titrated in 96-well tissue culture plate, in triplicates, with final concentrations ranging from 0 nm to 49 nM, in 7 nm increment. Briefly, each siRNA was dissolved in 200 μl of OPTI-MEM I Reduced Serum Medium (Gibco) mixed in equal volume of Lipofectamine RNAiMAx (Invitrogen, Carlsbad, CA, United States) with a final amount of 0.64 μl/well. siRNA and Lipofectamine were left for 5 min at room temperature for complexes to form, according to manufacturer’s instructions, and then were added to each well, mixed gently and incubated at 37°C for 48 h. At 48 h, cells were fixed in ice-cold 4% PFA. Rabbit polyclonal antibodies against human fibrinogen chain Aα antibody (20645-1-AP), fibrinogen chain Bβ antibody (16747-1-AP), and fibrinogen chain γ antibody (15841-1-AP) from Proteintech (Rosemont, IL, United States), were used. Donkey anti-Rabbit IgG H&L (Alexa Fluor^®^594) (A21207, Invitrogen, Carlsbad, CA, United States) were used as a secondary antibody. Immunofluorescence was processed as described above.

## Results

### Changes in Fibrinogen Chains Immunofluorescence Following SAH

**FIGURE 1:**
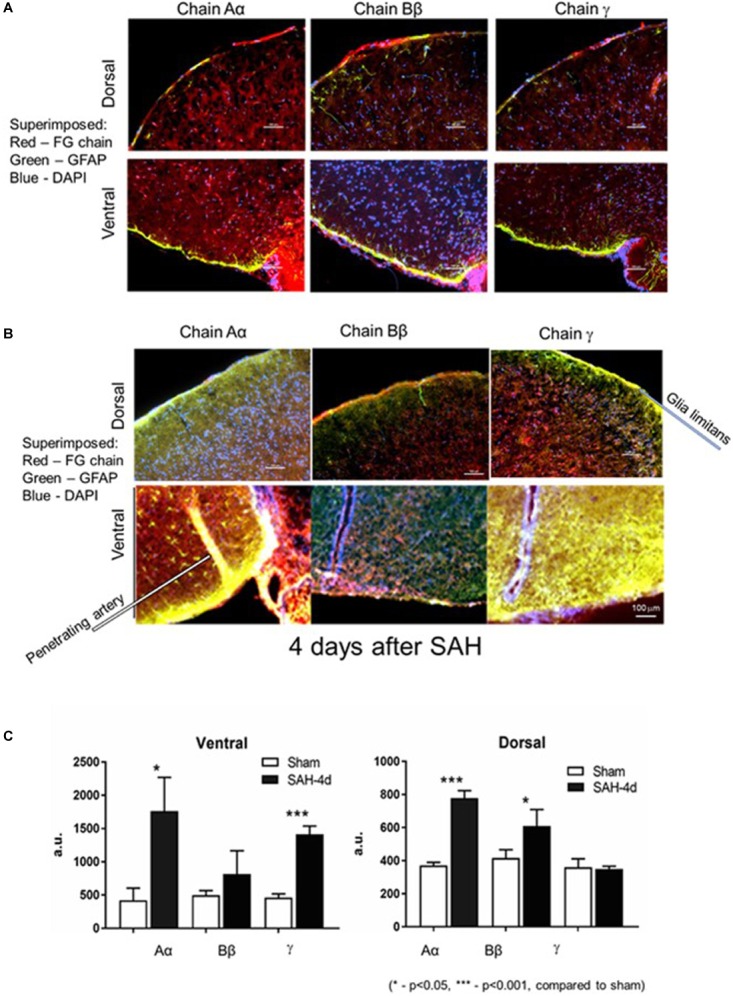
Distribution of fibrinogen chains (FG) Aα, Bβ, and γ in *glia limitans* and superficial layers of the brain dorsal and ventral surfaces **(A)** 4 days after surgery in sham hemorrhage animals and **(B)** 4 days after subarachnoid hemorrhage. **(C)** Changes in immunofluorescent intensity of the respective fibrinogen chains 4 days following the subarachnoid hemorrhage. Dorsal, dorsal surface of the brain; Ventral, ventral surface of the brain of the same animal. Red, fibrinogen chain; green, GFAP; blue, DAPI. a.u., arbitrary units. Bar – 100 μm. (3 animals/group; 5 ROI/animal, ^∗^*p* < 0.05).

In the first series of experiments we explored changes in the fibrinogen chains by immunofluorescence (IF) in the superficial layers of the parietal cortex and ventral surface of the mouse brain. We avoided areas on the ventral surface (especially) of the brain with visible signs of blood. Two groups of animals were compared: animals, in which the Circle of Willis was perforated 4 days before euthanasia and sham-operated animals in which no perforation was performed. Four days following SAH fibrinogen chains Aα IF associated with glia limitans and superficial layers of the ventral and dorsal superficial brain parenchyma increased 3.2 and 2.5 times (*p* < 0.05 and *p* < 0.01), respectively; fibrinogen chain Bβ increased by 3 times (*p* < 0.01) on the dorsal surface and fibrinogen chain γ increased by 3 times (*p* < 0.01) on the ventral surface compared to sham animals (Figure 1A–C). Importantly the changes in all three fibrinogen chains were un-coordinated suggesting differential distribution of fibrinogen chains in the dorsal and ventral superficial layers of the brain parenchyma.

**FIGURE 2:**
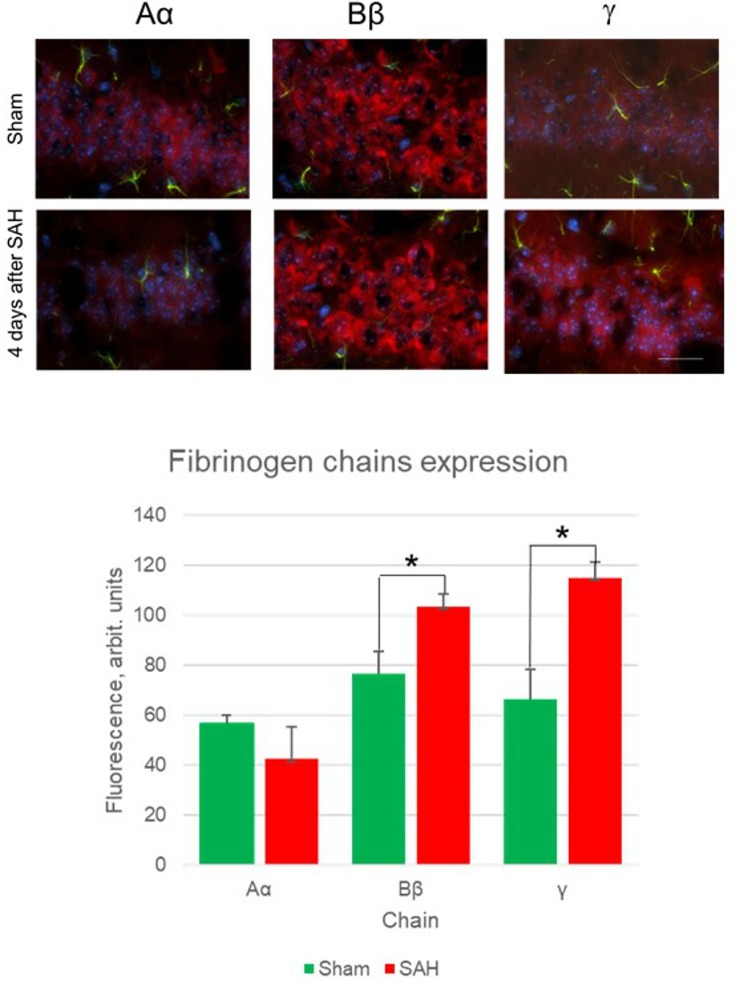
Fibrinogen chains expression changes 4 days after sham surgery and 4 days after SAH (upper panels) and quantification of immunofluorescence between sham animals and 4 days after SAH in hippocampal dentate gyrus. (5 animals/group, ^∗^*p*< 0.05, bar 100 μm).

Besides the increase in the presence of fibrinogen chains in the glia limitans and brain parenchyma, astrocytes and other cells also demonstrated the presence of fibrinogen chains immunoreactivity. Specifically, immunofluorescence of the cell-associated fibrinogen chains changed in the hippocampal dentate gyrus 4 days following the SAH (Figure 2).

### Human Normal Astrocytes

**FIGURE 3:**
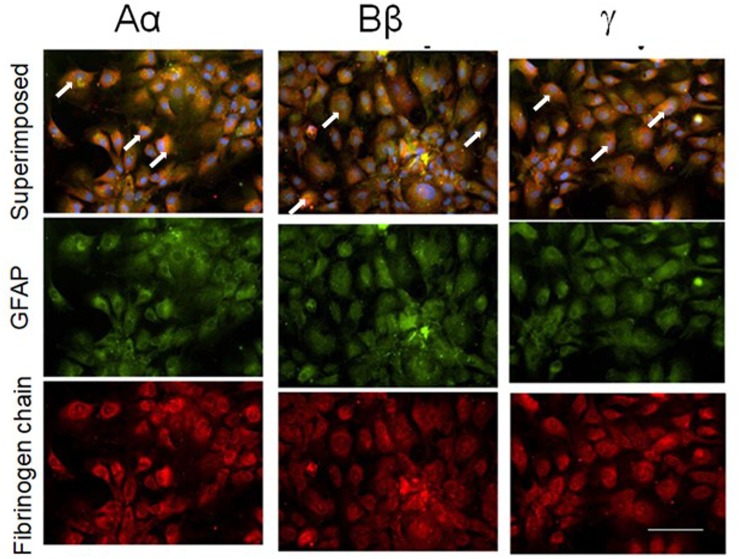
Normal human cultured astrocytes constitutively express all three fibrinogen chains immunofluorescence. Note co-localization (white arrows) of fibrinogen chains immunofluorescence and immunofluorescence of astrocytes labeled with GFAP antibodies. (bar – 100 μm).

**FIGURE 4:**
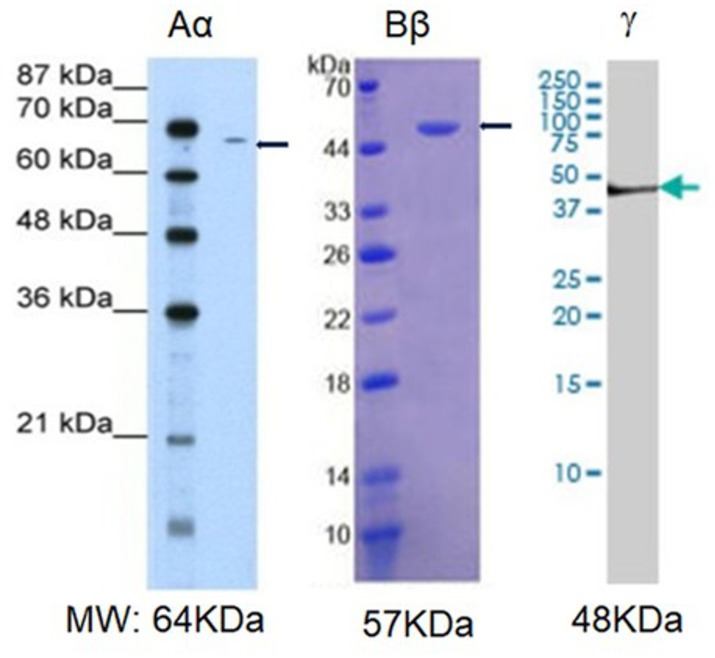
Western blot analysis of the fibrinogen immunopositive chains in normal human astrocytic culture. MW, established molecular weight of fibrinogen chains (Sharpe and Baskin, 2016).

Next, we explored whether NHA alone are capable of expressing fibrinogen chains. Indeed, immunochemical staining revealed that isolated astrocytes express immunoreactivity of all three fibrinogen chains (Figure 3). To determine whether the immunofluorescence was associated with the proteins we performed western blot analysis of the cell, and observed three bands with the molecular weights close to those reported for all three fibrinogen chains (McDonagh et al., 1972) (Figure 4).

### Effect of siRNA on the Expression of Fibrinogen Chains in Normal Human Astrocytes

**FIGURE 5:**
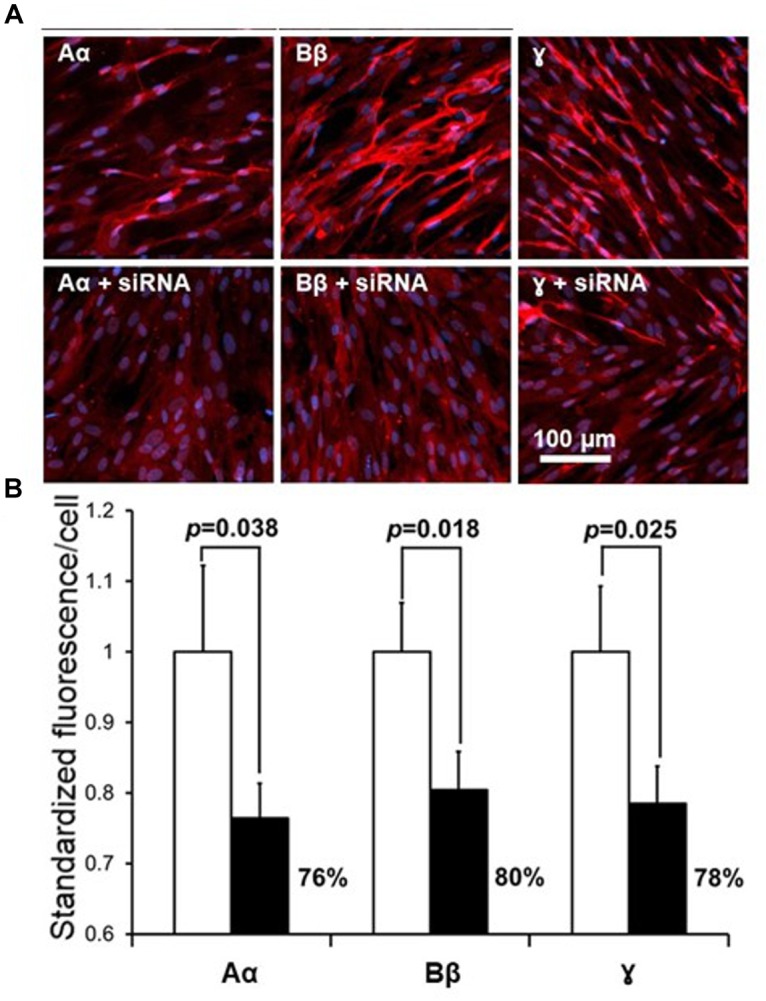
Effect of suppression of fibrinogen chains genes by siRNA in the culture of normal human astrocytes. **(A)** Representative fluorescent fields of view in control cultures (upper row) and in culture transfected with respective siRNA. Red fluorescence, fibrinogen chains; blue, DAPIB. Quantification of the suppression of fibrinogen chains expression by normal human astrocytic culture by siRNA. (**B**, Fluorescence was normalized by the determining the calculation of fluorescence/single cell, see Materials and Methods, all measurements were done in triplicate and three fields of view were taken in each well).

Following exploration of the fibrinogen chains proteins immunoreactivity levels in NHA we investigated silencing of the respective genes FGA, FGB and FGG using siRNA will suppress fibrinogen chains proteins immunofluorescence. Lipofection of the NHA with the specific siRNA, known to silence fibrinogen chains genes (Takezawa et al., 2013) we observed significant decrease of fibrinogen chains immunoreactivity in NHA. Specific siRNA decreased expression immunoreactive of fibrinogen chain Aα by 76%, fibrinogen Bβ by 80% and fibrinogen chain γ by 78%, respectively, at the concentration of siRNA of 49 nM (Figure 5). Decrease of the levels of fibrinogen chains after silencing of respective genes strongly suggest that that fibrinogen chains observed in NHA culture are of NHA origin and not resulting from external contamination, e.g., by bovine calf serum.

### Reaction to Biologically Significant Stimuli

**FIGURE 6:**
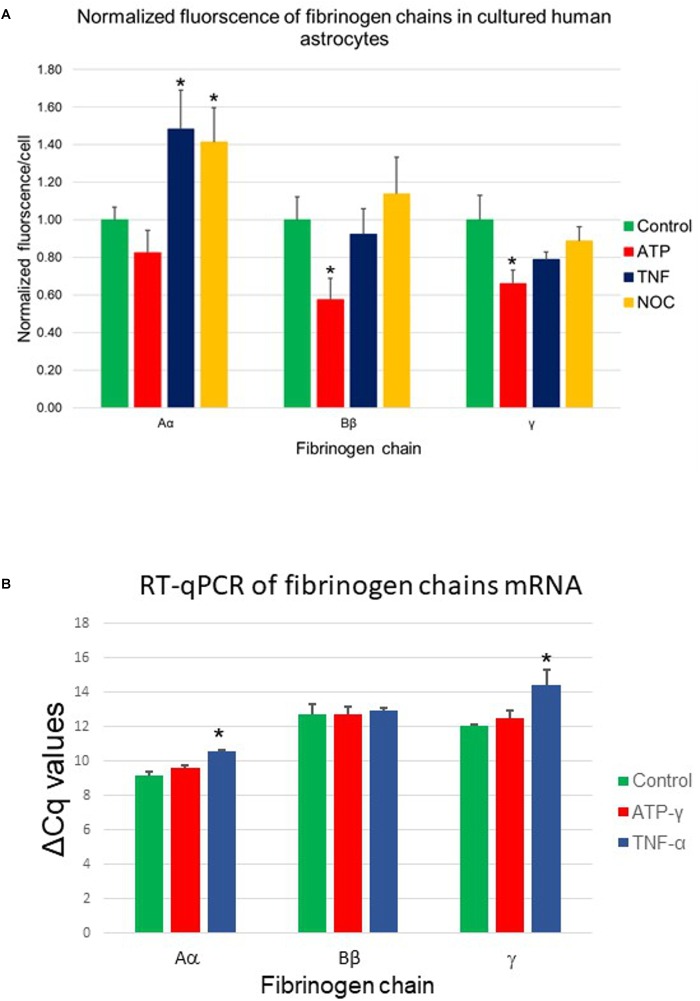
**(A)** Changes in fibrinogen chains immunofluorescence in normal human astrocytes differentially affected by 24-h exposure to pro-inflammatory stimuli. Aα, fibrinogen chain Aα; Bβ, fibrinogen chain Bβ; γ, fibrinogen chain γ; TNFα, tumor necrosis factor α; NOC, NO-donor and ATP-γ, stable ATP analog (three plates, each stimulus in triplicates, three fields of view). **(B)** Changes in fibrinogen chains mRNA (RT-qPCR) in normal human astrocytic culture in response to biologically significant stimuli. TNFα, tumor necrosis factor α; ATP-γ, stable ATP analog, ^∗^*p* < 0.05). (Three plates, each stimulus in triplicates).

In the following experiments, we explored whether expression of the fibrinogen chains can be modified by biologically important signals including (i) TNF-α, the cytokine associated with the acute phase reaction of inflammation, (ii) the slow releasing NO donor diethylenetriamine (NOC-18), which is known to participate in various processes, including the inflammatory response, and (iii) stable ATP analog, which is a danger-associated molecule, which initiates protective responses, including the inflammatory response. Expression of the fibrinogen chains differentially changed when exposed for 24 h to these biological stimuli (Table 1). Importantly, levels of immunoreactivity of each chain did not correlate. While fibrinogen chain Aα levels, determined by changes in immunofluorescence, increased by ∼50% (*p* < 0.01), fibrinogen chain Bβ levels decreased by almost 50% and did not change significantly to other stimuli. Fibrinogen chain γ also decreased in response to ATP administration. These experiments strongly indicate that fibrinogen chains differentially regulated by biologically significant stimuli (Figure 6A and Table 1).

**Table 1:** Changes in specific fibrinogen chains (FC) IF (arbitrary units/cell) in cultured human astrocytes in response to exposure to TNFα, NO-donor (NOC-18) and stable ATP analog (ATP-γ-S) (triplicates, 3 fields of view/well, m ± SEM).

	FC Aα	FC Bβ	FC γ
Control	1.00 ± 0.07	1.00 ± 0.12	1.00 ± 0.13
TNFα	1.48 ± 0.21^∗^	0.92 ± 0.14	0.79 ± 0.04
NOC-18	1.41 ± 0.18^∗^	1.14 ± 0.20	0.89 ± 0.08
ATP-γS	0.82 ± 0.12	0.58 ± 0.11^∗^	0.66 ± 0.07^∗^

*^∗^p < 0.01.*

### RT-qPCR in Astrocytes

The presence of the specific proteins suggested by immunohistochemistry would imply the presence of the specific mRNA if these proteins are being constitutively produced by cultured cells. To establish the presence of the transcription signal we performed RT-qPCR analysis of the presence of specific fibrinogen chain mRNA. Our results demonstrated the presence of the specific mRNA for each chain in the cultured astrocytes suggesting local transcription of the fibrinogen chains. Importantly, the levels of all three fibrinogens chains mRNA not only differed but were modulated differently by different biologically significant stimuli. These experiments strongly suggested that human astrocytes are capable of synthesizing fibrinogen chains, expression of which depends on the specific context (Figure 6B).

### Human Cortical Neurons

**FIGURE 7:**
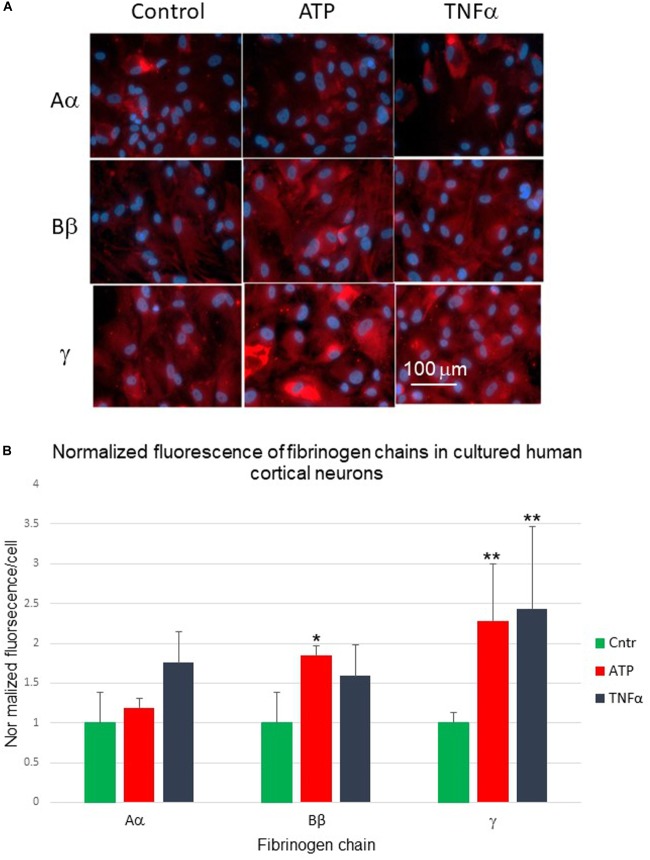
**(A)** Changes in fibrinogen chains immunofluorescence in normal human cortical neurons differentially affected by 24-h exposure to pro-inflammatory stimuli. Aα, fibrinogen chain Aα; Bβ, fibrinogen chain Bβ; γ, fibrinogen chain γ, **(B)** Normalized quantification of the changes of the fibrinogen chains fluorescence in normal human cortical neurons culture in response to biologically significant stimuli. (ATP, stable ATP-γ; TNFα, tumor necrosis factor α; ^∗^*p* < 0.05; ^∗∗^*p* < 0.01). (Three plates, each stimulus in triplicates).

Similarly, we explored constitutive expression and changes in fibrinogen chain immunoreactivities in the culture of human neurons. Human cortical neurons also demonstrated background expression of the fibrinogen chains. Exposure of cortical neurons to pro-inflammatory stimuli (TNF-α and ATP) changed the expression of fibrinogen chains as evaluated by immunofluorescence (Figure 7A). These changes were also differential and dependent on the type of stimulus. However, the pattern of the responses to similar stimuli differed from those observed in human astrocyte culture. Fibrinogen chain Bβ level increased by 85%, at the same time fibrinogen chain γ increased by over 100% in response to administration of ATP in neurons, while it decreased in response to ATP in astrocytes (Figure 7B and Table 2).

**Table 2:** Changes in specific fibrinogen chains (FC) IF (arbitrary units/cell) in cultured human cortical neurons in response to exposure to TNFα, and stable ATP analog (ATP-γ-S) (triplicates, 3 fields of view/well, m ± SEM).

	FC Aα	FC Bβ	FC γ
Control	1.00 ± 0.38	1.00 ± 0.32	1.00 ± 0.13
TNFα	1.76 ± 0.39	1.59 ± 0.56	2.43 ± 1.03
ATP-γS	1.19 ± 0.12	1.85 ± 0.54^∗^	2.28 ± 0.72^∗^

*^∗^p < 0.01.*

**FIGURE 8:**
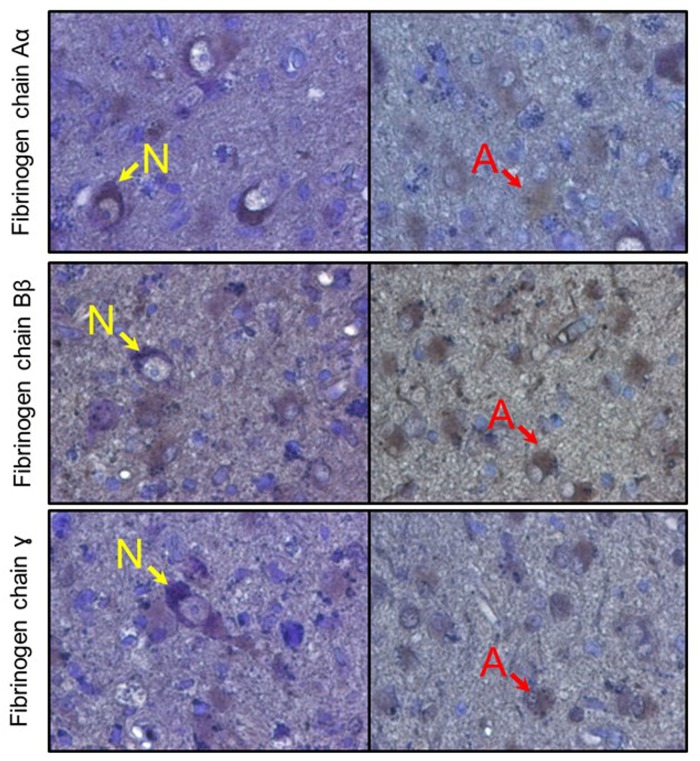
Immunoreactive detection (DAB + hematoxylin) of fibrinogen chains in human brain samples (amygdala). Rows represent respective fibrinogen chains. N, indicate neurons, A, indicate astrocytes.

### Human Tissue

After establishing that cultured human neurons and astrocytes are capable of expressing the three fibrinogen chains and that increases in fibrinogen chain-associated immunofluorescence in the mouse brain, including their intracellular localization, we analyzed whether fibrinogen chains are present in human brain tissue. We analyzed brain parenchyma samples obtained from different human brain structures (amygdala, hypothalamus) and observed that single cell-associated fibrinogen chain–associated immunofluorescence can be observed in human brain cells that displayed neuronal or astrocyte morphology, while there was no fibrinogen chain-related immunoreactivity in the extracellular space (Figure 8).

## Discussion

We demonstrate, for the first time, that astrocytes and neurons express fibrinogen chains suggesting potential presence of endogenous to the brain fibrinogen chains. Along with others we observed fibrin deposition in the areas remote to hemorrhage following the SAH (Suzuki et al., 1977; Golanov et al., 2017). It is conceivable that in spite of the remote location of the fibrin deposition in the subarachnoid space following the SAH fibrinogen and associated coagulation factors of the extrinsic pathway diffuse there along with CSF and polymerize through the extrinsic pathway as it is known that the bounding glia limitans contains high levels of TF that can initiate fibrinogen conversion to fibrin (Golanov et al., 2017). In our described experiments cells of the glia limitans also demonstrated high levels of fibrinogen chains, which conceivably could be endocytosed from the CSF. Similarly, increased levels of fibrinogen chains in the parenchyma following the SAH could be due to changes in the permeability barrier of cerebral microvessels following the SAH even in the remote sites, where no blood elements were observed (Davalos and Akassoglou, 2012; Petersen et al., 2018). However, in our experiments changes in the levels of fibrinogen chains were not correlated. Moreover, the fibrinogen chains were also accumulated in the intraparenchymal cells. These observations suggest that fibrinogen chains at least partially can be of local origin, i.e., generated by the glia limitans and other non-vascular brain cells. Furthermore, fibrinogen chain immunoreactivities were also detectable in the hippocampal dentate gyrus, where no blood was observed.

We also explored NHA and cortical human neuron cultures for the presence of the three fibrinogen chains. Immunohistochemistry revealed the intracellular presence of all three fibrinogen chains in human astrocytes and neurons. It can be argued that cultured cells endocytosed fibrinogen from the FBS-containing culture media. Western blot revealed presence of the specific fibrinogen chain proteins with the appropriate molecular weight. Low levels of fibrinogen in calf fetal serum and washing the cells before fixation (Ayache et al., 2006) decrease probability of cell “contamination” with the exogenous fibrinogen chains. However, this does not completely exclude origination of intracellular fibrin chains from the serum fibrinogen. Corroborating are our findings that the astrocyte cultures express the specific mRNAs for each chain, the observation which supports the hypothesis of an intracellular origin of the fibrinogen chains.

Allen atlas also demonstrates possible wide spread expression of fibrinogen chain Aα^1^, presence of expression, though much more limited, of fibrinogen chains Bβ^2^ and γ^3^ (Morris et al., 2010). Expression of fibrinogen γ in the brain was demonstrated earlier (Haidaris and Courtney, 1990). These data provide additional support for the possible fibrinogen chains expression by the mouse brains.

Moreover, immunohistochemically determined levels of fibrinogen chains varied differentially in response to exposure to biologically significant signals. Importantly, responses, for example, of fibrinogen chain γ differed in neurons and astrocytes to the same signal, ATP-γS. These data suggest that the expression of the fibrinogen chains in cultured human neurons and astrocytes is intrinsic and regulated independently of each other.

Taken together these data strongly suggest the possibility that all three fibrinogen chains can be expressed in the brain, by neurons and astrocytes.

Fibrinogen chains appears in numerous brain pathologies: multiple sclerosis, Alzheimer’s disease, EAE, posttraumatic demyelination as well as possible involvement in depression (Davalos and Akassoglou, 2012; Chiam et al., 2015; Petersen et al., 2018). In all cases it was suggested that the cause of appearance of fibrinogen in the brain is a “leaky” BBB. Indeed, in most cases the cerebral microvessel permeability barrier is abnormal and allows fibrinogen to enter the brain (Davalos and Akassoglou, 2012; Petersen et al., 2018). Because of fibrin deposits appear in Alzheimer’s disease often localized near vessels a role for an altered blood-brain barrier has been suggested (Ryu and McLarnon, 2009; Sagare et al., 2013). However, there are also fibrin deposits in the neuropil without clear BBB disruption (Bien-Ly et al., 2015), which might occur due to the local production of fibrinogen.

Notably, fibrinogen chains as independent units are capable of interacting with various molecules (Weisel, 2005). Fibrinogen chains Aα and γ are capable of interaction with the various integrins such as αMβ2, αIIIβV mostly through their fibrinogen-related domain Arg–Gly–Asp (RGD) (Smith et al., 1990; Suehiro et al., 2000; Lishko et al., 2002; Yakubenko et al., 2002; Podolnikova et al., 2003; Ugarova et al., 2003; Lilja and Ivaska, 2018). The important role of integrins in the regulation of extracellular matrix and synaptic activity is well-established (Wu and Reddy, 2012; Park and Goda, 2016; Lilja and Ivaska, 2018). It is conceivable that fibrinogen chains Aα and γ can play modulatory roles through their interaction with various integrins in neuronal activity and other processes. Longer fibrinogen-related domain (Doolittle et al., 2012) of fibrinogen chain Bβ and fibrinogen chain γ also interacts with TLR-4 (Zuliani-Alvarez et al., 2017) potentially playing important roles in innate immunity and inflammation (Trotta et al., 2014). Fibrinogen chain Aα also interacts with fibronectin and TGF-β playing roles in the neuroinflammation (Engvall et al., 1978; Schachtrup et al., 2010; Webster et al., 2017). Fibrinogen chain Bβ can interact with VE-cadherin and β-amyloid (Yakovlev and Medved, 2009; Ahn et al., 2010). Fibrinogen chain γ is capable of interaction with ICAM-1 (Altieri et al., 1995). As our experiments demonstrated, fibrinogen chains differentially respond to biologically significant and pro-inflammatory signals. It is conceivable that intrinsic fibrinogens chains play roles in the brain pathology and brain responses to various stimuli. Fibrin(ogen) itself is known to exert damaging effects on brain tissue (Ryu et al., 2009). However, the origin of separate fibrinogen chains could be different, extrinsic and intrinsic. It is conceivable that intrinsic fibrinogen chains can exert pleiotropic effects modulating various processes in norm and pathology.

## Conclusion

Our data strongly suggest that neurons and astrocytes are capable of production of fibrinogen chains, which may be involved in various normal processes and brain pathology. Additional experiments are necessary to establish (a). whether cell/structure-specific fibrinogen chain genes knock-outs/knock-ins would have specific phenotype; (b) what physiological/behavioral effects fibrinogen chains alone would exercise being introduced directly into the brain; (c) to what extent they are capable to modify synaptic functions; (d) whether they play specific roles in various brain pathologies. The possibility that fibrinogen chains may have intrinsic to the brain origin open new intriguing venues for our understanding of established brain pathologies, including SAH.

## Contribution to the Field Statement

Discovery of the expression of all fibrinogen chains by astrocytes and neurons in the brain *in vivo* and *vitro* suggests that fibrinogen observed in various neuropathological conditions can be of endogenous to the brain origin. Demonstration that the fibrinogen chains are differentially regulated by pro-inflammatory stimuli suggests that fibrinogen chains may play different role in brain functioning and brain response to the neuroinflammatory processes occurring during various neurological diseases. Traditionally it is thought that fibrinogen enters brain/subarachnoid space through damaged blood brain barrier. We demonstrate for the first time that mouse and human astrocytes and neurons express fibrinogen chains suggesting potential presence of endogenous to the brain fibrinogen chains differentially changing to biologically significant stimuli. SAH is followed by increased expression of fibrinogen chains associated with glia limitans remote from the hemorrhage. We conclude that brain astrocytes and neurons are capable of production of fibrinogen chains, which may be involved in various normal and pathological processes in the brain.

## Data Availability

The datasets generated for this study are available on request to the corresponding author.

## Author Contributions

EG, MS, and AR-G conceived, planned, and carried out the experiments, processed the data, and wrote the manuscript. GDZ conceived and planned the experiments, analyzed the data, and edited the manuscript. DB and GB planned the experiments, interpreted the data, and edited the manuscript.

## Conflict of Interest Statement

The authors declare that the research was conducted in the absence of any commercial or financial relationships that could be construed as a potential conflict of interest.

## Footnotes

1 http://mouse.brain-map.org/experiment/show/68442907

2 http://mouse.brain-map.org/experiment/show/68638095

3 http://mouse.brain-map.org/gene/show/63741
